# Direct Oral Anticoagulants for Cancer-Associated Venous Thromboembolism

**DOI:** 10.1007/s11912-023-01428-y

**Published:** 2023-06-06

**Authors:** Marta Masini, Matteo Toma, Paolo Spallarossa, Italo Porto, Pietro Ameri

**Affiliations:** 1 Department of Internal Medicine, University of Genova, Viale Benedetto XV, 6 - 16132 Genoa, Italy; 2 Cardiovascular Disease Unit, IRCCS Ospedale Policlinico San Martino, Genoa, Italy

**Keywords:** Anticoagulation, Thrombosis, Embolism, Cancer, Cardio-oncology

## Abstract

**Purpose of Review:**

To present the randomized controlled trial (RCT) evidence and highlight the areas of uncertainty regarding direct oral anticoagulants (DOAC) for cancer-associated venous thromboembolism (CAT).

**Recent Findings:**

In the last years, four RCTs have shown that rivaroxaban, edoxaban, and apixaban are at least as effective as low-molecular-weight heparin (LMWH) for the treatment of both incidental and symptomatic CAT. On the other hand, these drugs increase the risk of major gastrointestinal bleeding in patients with cancer at this site. Another two RCTs have demonstrated that apixaban and rivaroxaban also prevent CAT in subjects at intermediate-to-high risk commencing chemotherapy, albeit at the price of higher likelihood of bleeding. By contrast, data are limited about the use DOAC in individuals with intracranial tumors or concomitant thrombocytopenia. It is also possible that some anticancer agents heighten the effects of DOAC via pharmacokinetic interactions, up to making their effectiveness-safety profile unfavorable.

**Summary:**

Leveraging the results of the aforementioned RCTS, current guidelines recommend DOAC as the anticoagulants of choice for CAT treatment and, in selected cases, prevention. However, the benefit of DOAC is less defined in specific patient subgroups, in which the choice of DOAC over LMWH should be carefully pondered.

## Introduction

In spite of major advances in oncological care, cancer-associated venous thromboembolism (CAT), including deep venous thrombosis (DVT) and pulmonary embolism (PE), remains highly prevalent in cancer patients, with substantial health and social costs [1].

It has been estimated that subjects with cancer face a 4- to 6- sixfold increased risk of venous thromboembolism (VTE) compared with the general population [2]. Tumors may initiate the coagulation cascade directly, by producing pro-coagulant molecules, or via secretion of mediators like pro-inflammatory cytokines, which activate the endothelium and stimulate platelet aggregation. Malignant cells may also invade venous vessels, damage the vascular wall, and cause blood stasis with ensuing thrombosis [3]. Furthermore, anti-neoplastic therapies may trigger VTE: this is the case, for instance, with thalidomide and lenalidomide, BCR-ABL tyrosine kinase inhibitors, and agents targeting the vascular endothelial growth factor receptor [4]. Finally, oncological patients often have general risk factors for VTE, such as prolonged immobilization and indwelling central venous catheters.

## From Low-Molecular-Weight Heparin to Direct Oral Anticoagulants for Cancer-Associated Venous Thromboembolism

Subcutaneous low-molecular-weight heparin (LMWH) was the recommended anticoagulant for CAT until a few years ago [5–7].

The use of this drug for CAT is grounded in a limited number of studies that enrolled in total around 2000 subjects [8–12], with two open-label randomized controlled trials (RCTs) with blinded outcome adjudication with dalteparin and tinzaparin accounting for 78% of all patients [8, 9]. Overall, the rate of recurrent CAT was consistently, but most often not significantly, lower with LMWH than with vitamin K antagonists (VKA); by contrast, the frequency of bleeding events was discordant across investigations (Table 1).

**Table 1:** Randomized controlled trials of anticoagulant therapy for cancer-associated venous thromboembolism

**Study** **(Ref. #)**	**Duration**	**Tested LMWH**	**Recurrent VTE**		**Major bleeding**	
			**LMWH**	**VKA**	**LMWH**	**VKA**
CLOT (8)	6 months	Dalteparin	27/336 (8%)	53/336 (15.8%)*	19/338 (5.6%)	12/335 (3.6%)
CATCH (9)	6 months	Tinzaparin	31/449 (6.9%)	45/451 (10%)	12/449 (2.7%)	11/451 (2.4%)
CANTHANOX (10)	3 months	Enoxaparin	2/71 (2.8%)	3/75 (4%)	5/71 (7%)	12/75 (16%)
ONCENOX (11)	6 months	Enoxaparin	4/61 (6.6%)	3/30 (10%)	6/67 (8.9%)	1/34 (2.9%)
LITE (12)	3 months	Tinzaparin	18/369 (4.9%)	21/368 (5.7%)	10/144 (6.9%)	13/146 (8.9%)
**Study** **(Ref. #)**	**Duration**	**Tested DOAC**	**Recurrent VTE**		**Major bleeding**	
			**DOAC**	**dalteparin**	**DOAC**	**dalteparin**
SELECT-D (29)	6 months	Rivaroxaban	8/203 (3.9%)	18/203 (8.9%)*	11/203 (5.4%)	6/203 (3%)
Hokusai VTE Cancer (30)	Up to 9 months	Edoxaban	41/522 (7.9%)	59/524 (11.3%)	36/522 (6.9%)	21/524 (4%)*
ADAM VTE (31)	6 months	Apixaban	0/145 (0%)	2/142 (1.4%)	1/145 (0.7%)	9/142 (6.3%)
Caravaggio (32)	6 months	Apixaban	32/576 (5.6%)	46/579 (7.9%)	22/576 (3.8%)	23/579 (4%)

The upper part of the table shows the frequency of recurrent venous thromboembolism (VTE) and major bleeding in randomized controlled trials comparing different types of low-molecular-weight heparin (LMWH) with vitamin K antagonists (VKA), while the lower part presents the same outcomes in trials evaluating direct oral anticoagulants (DOAC) vs. dalteparin. Event rates are expressed as total number divided by treated patients, as published

^*^Indicates significant difference for the corresponding hazard ratio

It is noteworthy that persistence on full-dose LMWH is low, due to inconvenience of subcutaneous administration for individuals who frequently already have to bear demanding therapies, fear of bleeding, and, in some countries, financial concerns [13]. Among 52,911 outpatients with newly diagnosed cancer who developed VTE and were prescribed LMWH between 2009 and 2014 in the USA, only 13% maintained such treatment over the following 6 months [14].

Direct oral anticoagulants (DOAC) were developed to overcome the erratic pharmacokinetics and pharmacodynamics of VKA, which blunt the coagulation system indirectly, by interfering with vitamin K–dependent synthesis of coagulation factors II, VII, IX, and X in the liver. As such, the effect of VKA is delayed, typically by 12 to 72 h, and influenced by genetically determined activity of hepatic enzymes, hepatocyte function, and endogenous and dietary vitamin K levels [15]. Instead, DOAC directly inhibit coagulation factors Xa (edoxaban, rivaroxaban, and apixaban) or IIa (dabigatran). The onset of action of these medications is rapid, the relationship between systemic drug concentrations and degree of anticoagulation is predictable, and it is possible to use fixed doses without laboratory monitoring [16]. These characteristics lead to a better efficacy-safety profile as compared with VKA, which has made DOAC the oral anticoagulants of choice for a wide range of indications, including VTE.

The RCTs evaluating DOAC for VTE were conducted in unselected populations comprising small subgroups with active cancer [17–21]. Until recently, analyses of these subgroups [22–25] and retrospective studies [26–28] represented the evidence supporting the prescription of DOAC for CAT. DOAC appeared to be associated with fewer recurrences of VTE than LMWH or VKA and variable rates of bleeding. However, these data are flawed by methodological limitations, such as selection and confounding bias, comparison with either VKA or LMWH, and lack of information about cancer stage and treatment.

In the last years, the results of a series of RCTs have consolidated the indication of DOAC for CAT treatment, as well as prevention in cancer patients at high risk of VTE. On the other hand, there are still areas of uncertainty regarding the use of DOAC for CAT.

## Randomized Controlled Trials Evaluating Direct Oral Anticoagulants for Treatment of Cancer-Associated Venous Thromboembolism

Four phase 3, open-label, multicenter RCTs assessed DOAC vs subcutaneous dalteparin in patients with CAT (Table 1).

**Fig. 1:**
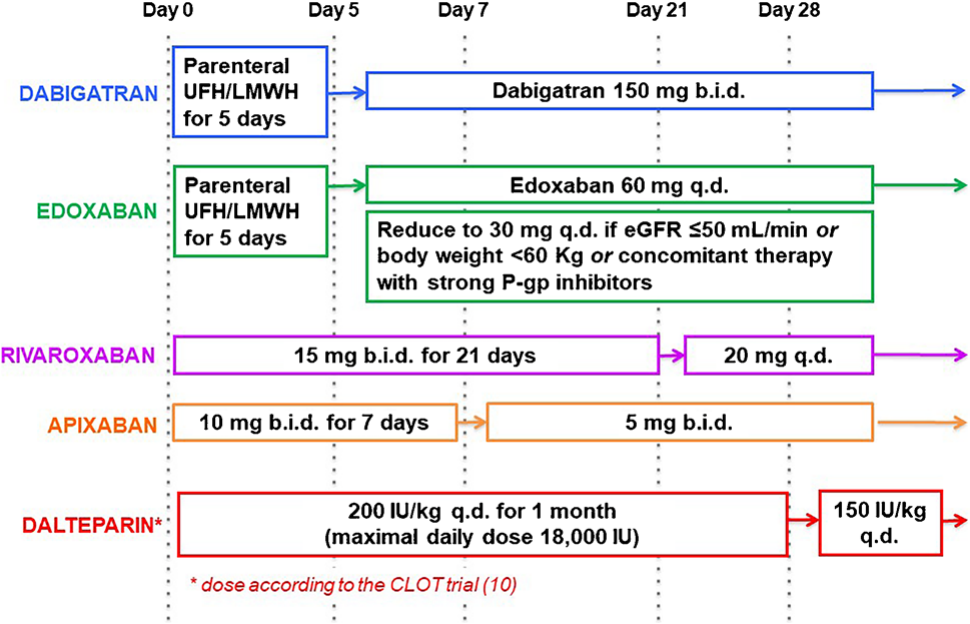
Therapeutic anticoagulation schemas for the treatment of cancer-associated venous thromboembolism. b.i.d., bis in die; eGFR, estimated glomerular filtration rate, as calculated by the Cockcroft-Gault formula; LMWH, low-molecular-weight heparin; P-gp, P glycoprotein; q.d., quaque die; UFH, unfractionated heparin

The earliest study, Comparison of an Oral Factor Xa Inhibitor with Low Molecular Weight Heparin in Patients with Cancer with Venous Thromboembolism (SELECT-D), compared rivaroxaban and dalteparin in 406 subjects with cancer and symptomatic or incidental PE or symptomatic DVT [29.••••]. Rivaroxaban was administered at the dosage of 15 mg twice daily for 3 weeks followed by 20 mg daily, while dalteparin treatment consisted in 200 IU per kilogram of body weight once daily for the first month, and then 150 IU per kilogram once daily (Fig. 1). The primary outcome was recurrence of VTE over 6 months and was significantly less frequent in the rivaroxaban than in the dalteparin arm (3.9% vs. 8.9%; HR 0.43, 95% CI 0.19–0.99). Major bleeding was non-significantly more frequent with rivaroxaban than with dalteparin (5.4% vs. 3%; HR 1.83, 95% CI 0.68–4.96). Conversely, clinically relevant non-major bleeding was significantly increased by rivaroxaban (12.3% vs. 3.4%; HR 3.76, 95% CI 1.63–8.69). Risk of bleeding was highest in the subgroup with gastroesophageal tumors [29.••••].

The Edoxaban for the Treatment of Cancer-Associated Venous Thromboembolism (Hokusai VTE Cancer) trial evaluated edoxaban vs dalteparin in 1050 patients with symptomatic or incidental VTE and cancer other than basal cell and squamous cell skin cancer, which had been diagnosed within the previous 2 years [30.••••]. Edoxaban was initiated after at least 5 days of parenteral anticoagulation with intravenous unfractionated heparin or subcutaneous LMWH and was given at the dose of 60 mg once daily. This dosage was reduced to 30 mg once daily in case of estimated glomerular filtration rate < 50 mL/min/1.73 m^2^, body weight < 60 kg, or concomitant use of inhibitors of P-glycoprotein (P-gp, also see next section). Dalteparin was given with the same schema as in SELECT-D. Therapy lasted for at least 6 and up to 12 months, and minimum follow-up was 9 months. The primary end-point was a composite of recurrent symptomatic or incidental VTE and major bleeding, i.e., overt bleeding associated with a drop in hemoglobin concentration of ≥ 2 g/dl or the need of transfusion of ≥ 2 units of blood, happening at a critical site, or contributing to death. This primary outcome occurred in 12.8% of edoxaban-treated patients and 13.5% of those assigned to dalteparin (HR 0.97, 95% CI 0.70–1.36). Secondary analyses revealed a trend for lower rate of VTE recurrence (7.9% vs. 11.3%; HR 0.71, 95% CI 0.48–1.06) and a significantly higher risk of major bleeding (6.9% vs. 4%; HR 1.77, 95% CI 1.03–3.04) with edoxaban [30.••••]. Again, major bleeding was mostly upper gastrointestinal.

Apixaban was first investigated as an alternative to dalteparin in an investigator-initiated, US-based, multicenter RCT, Apixaban or Dalteparin in Reducing Blood Clots in Patients With Cancer Related Venous Thromboembolism (ADAM VTE) [31.••••]. Three hundred patients received apixaban (10 mg twice daily for the first 7 days, followed by 5 mg twice daily) or dalteparin (same dosage as in SELECT-D and Hokusai VTE Cancer). The study was designed to test the superiority of apixaban over dalteparin in reducing the primary outcome of major bleeding up to 6 months, which occurred at a rate of 0% with apixaban and 1.4% with dalteparin (HR and 95% CI not estimable). The secondary endpoint of any thromboembolism (DVT, PE, and arterial thromboembolism) was observed in 0.7% and 6.3% of individuals assigned to apixaban and dalteparin, respectively (HR 0.09, 95% CI, 0.01–0.78) [31.••••].

Subsequently, the multinational, non-inferiority Apixaban for the Treatment of Venous Thromboembolism in Patients With Cancer: A Prospective Randomized Open Blinded End-Point (Probe) Study (CARAVAGGIO) randomized 576 subjects with cancer and symptomatic or incidental VTE to apixaban or dalteparin (same doses as above) [32.••••]. The primary outcome of recurrent VTE at 6 months was faced by 5.6% of patients in the apixaban group and 7.9% of those assigned to dalteparin (HR 0.63, 95% CI 0.37–1.07), while the rates of major bleeding were 3.8% and 4%, respectively (HR 0.82, 95% CI 0.4–1.69) (Table 1).

In summary, these RCTs showed that DOAC are at least as effective as dalteparin in protecting against CAT recurrence. Nevertheless, treatment with edoxaban and rivaroxaban took the toll of a higher risk of bleeding, primarily in patients with gastrointestinal malignancies. This side effect has been ascribed to the direct action of DOAC within the gastrointestinal tract, but it was not observed with apixaban [32.••••].

At least half of the participants in these RCTs had metastatic cancer, and no heterogeneity was found between subjects with or without metastasis [29.••••, 30.••••, 31.••••, 32.••••]. Additional sub-analyses confirmed the efficacy and safety profile of DOAC vs dalteparin for CAT across different tumor sites and stages, with the aforementioned exception of enhanced bleeding in individuals with gastrointestinal cancer receiving edoxaban or rivaroxaban [33, 34].

## Randomized Controlled Trials Evaluating Direct Oral Anticoagulants for Prevention of Cancer-Associated Venous Thromboembolism

Two RCTs addressed the use of DOAC to prevent CAT in subjects who start chemotherapy and are at intermediate-to-high risk for VTE, as defined by a Khorana score ≥ 2. Previously, LMWH had been demonstrated to be superior to placebo for VTE prevention in ambulatory oncological patients receiving chemotherapy [35.••••, 36.••••], especially for pancreatic cancer [37].

The Apixaban for the Prevention of Venous Thromboembolism in Cancer Patients (AVERT) phase 2 RCT compared apixaban with placebo [35.••••], and the Rivaroxaban for Thromboprophylaxis in High-Risk Ambulatory Patients with Cancer (CASSINI) phase 3 RTC compared rivaroxaban with placebo [36.••••]. In both studies, follow-up was up to 180 days and the main safety outcome was major bleeding, while the primary efficacy outcome was VTE in AVERT and VTE and death from VTE in CASSINI.

In a modified intention-to-treat analysis including 563 out of 574 subjects randomized in AVERT, VTE occurred significantly less frequently with apixaban than with placebo (4.2% vs. 10.2%, HR 0.41, 95% CI 0.26–0.65), but the opposite happened for major bleeding (3.5% vs. 1.8%; HR 2.0, 95% CI 1.01–3.95). In CASSINI, the primary composite efficacy end-point as well as major bleeding were less frequent—albeit not to a significant extent—in patients randomized to rivaroxaban than placebo (6.0% vs. 8.8%, HR 0.66, 95% CI 0.40–1.09 for the efficacy endpoint; and 2.6% vs. 6.4%, HR 1.96, 95% CI 0.59–6.49 for major bleeding).

When considering only the treatment period (i.e., the time interval during which the study drug was actually taken), major bleeding was no longer significantly different between the apixaban and placebo groups of AVERT (2.1% vs. 1.1%; HR 1.89, 95% CI 0.39–9.24) and the primary end-point of CASSINI occurred significantly less often in rivaroxaban than placebo treated patients (2.6% vs. 6.4%; HR 0.40, 95% CI 0.20–0.80) [35.••••, 36.••••].

It is important to note that both apixaban and rivaroxaban were tested in these RCTs at doses different from the ones investigated in VTE trials, respectively 2.5 mg twice daily and 10 mg daily.

## Open Issues in the Management of Cancer-Associated Venous Thromboembolism with Direct Oral Anticoagulants

### Pharmacological Interactions

Dabigatran is the DOAC with the most extensive renal clearance (about 80%), while rivaroxaban and apixaban are substantially metabolized by the cytochrome P450, CYP3A4 [38, 39]. All DOAC are excreted in the intestine and to a lesser extent in the kidney via P-gp, so inhibitors of this transporter may increase DOAC plasma concentrations.

Based on predicted or known modulation of CYP3A4 and P-gp, DOAC should be cautiously used in case of co-treatment with many anti-tumor drugs, such as doxorubicin, abiraterone, enzalutamide, imatinib, and sunitinib [38]. Nonetheless, no signal of lower efficacy or safety was observed in the subgroups on active anti-cancer therapy in RCTs of DOAC for CAT [29.••••, 30.••••, 31.••••, 32.••••]. The dosage of edoxaban is halved when patients are taking major P-gp inhibitors (Fig. 1), and this strategy likely prevents most harmful consequences of the pharmacokinetic interaction between edoxaban and other medications acting on P-gp [40]. It is remarkable that the comparative efficacy and safety of apixaban and dalteparin was not different in participants in CARAVAGGIO who were or were not concomitantly given anti-cancer agents (around 60% and 40%, respectively) [41], even though therapy with P-gp modulators is not a criterion to modify the dose of apixaban.

### Incidental Cancer-Associated Thromboembolism

CAT may be incidentally detected during imaging exams performed for unrelated reasons, such as tumor staging [42]. Retrospective analyses indicate that the rate of recurrent VTE after a first incidental diagnosis of CAT is similar to the one observed after symptomatic CAT, encouraging the institution of anticoagulant therapy [43, 44]. As many as 30% of patients in Hokusai VTE Cancer, 50% in SELECT-D, and 20% in CARAVAGGIO had incidental CAT at enrollment [29.••••, 30.••••, 32.••••]. This presentation was more common for PE than for DVT [45, 46]. In agreement with observational studies, the rates of clinical adverse outcomes were substantial in both incidental and symptomatic CAT arms. Bleeding was particularly frequent in subjects given DOAC or dalteparin for clinically silent CAT, implying that the balance between the anti-thrombotic and pro-hemorrhagic activity of anticoagulants is critical in this setting.

A peculiar type of asymptomatic CAT is the one involving venous catheters. In an open-label investigation in which 70 subjects with central venous catheter-related VTE received rivaroxaban for 12 weeks, the central venous line was always preserved and the rate of recurrent VTE was 1.43%, but there was 1 episode of fatal PE and 9 (12.9%) patients experienced bleeding events [47].

### Prolonged Anticoagulation

Two prospective, multicenter, single-arm studies, DALTECAN and TICAT, collected long-term data in patients with active cancer and VTE treated with dalteparin for a maximum of 1 year or tinzaparin for 1 year, respectively [48, 49]. In both, the frequency of recurrent VTE and major bleeding was highest in the first months and declined thereafter. However, only 32% of participants in DALTECAN and 55% of those enrolled in TICAT completed 12 months of treatment. In phase 3 RCTs, DOAC therapy lasted 6–12 months [29.••••, 30.••••, 32.••••].

At present, the choice of prolonging anticoagulation beyond 6–12 months should be tailored considering the severity of CAT and the risk of bleeding, but also tumor- and patient-related factors [50, 51]. For instance, gastrointestinal and genitourinary cancer are associated with more VTE and bleeding events on anticoagulant therapy, including DOAC [29.••••, 30.••••], and VTE recurs more often with locally advanced or metastatic than localized cancer [31.••••]. Furthermore, a recent analysis of a subset of 652 patients with cancer-associated PE in Hokusai VTE Cancer showed that worse performance status at follow-up was associated with both anticoagulation discontinuation and heightened risk of VTE recurrence and major bleeding [52].

### Intracranial Tumors

Intracranial neoplasms, either primary or metastatic, are matter of extreme concern when starting anticoagulation because of the possibility of cerebral hemorrhage [53, 54]. Few subjects with intracranial tumors were recruited in Hokusai VTE Cancer, SELECT-D, and ADAM-VTE, and none in CARAVAGGIO. Retrospective studies showed that DOAC do not portend an increased rate of major bleeding compared with LMWH in patients with primary brain tumors or brain metastases, although intracranial hemorrhage is more likely with the latter ones [55–58].

### Thrombocytopenia

Anticoagulation is contraindicated when platelets are < 25 × 10^9^/L [59]. When platelets are between 25 and 50 × 10^9^/L, LMWH should be used since data with DOAC in this context are scarce [59]. In fact, the platelet count threshold in RCTs of DOAC for CAT ranged from 50 × 10^9^/L (Hokusai VTE Cancer and ADAM VTE) to 75 × 10^9^/L (CARAVAGGIO) to 100 × 10^9^/L (SELECT-D) [25–28].

LMWH is also preferred over DOAC when platelets are > 50,000 × 10^9^/L, but with possibility of a further decrease in the subsequent days [60.••••]. Similarly, prophylactic-dose LMWH, and not DOAC, may be considered to prevent CAT in individuals with thrombocytopenia, with close monitoring of the platelet count [60.••••].

In presence of acute VTE or high thrombotic risk, or when there is a history of recurrent or progressive thrombosis, transfusions may be performed to raise the platelet count above 50 × 10^9^/L and, therefore, make full-dose anticoagulation feasible. Conversely, if the thrombotic risk is low, LMWH can be given at a dosage reduced by 25–50%, and the dose can be adjusted to 50% of the normal in case of subacute or chronic VTE [61]. Modified-dose anticoagulation has been shown to be safe in patients with active malignancy, acute VTE and a platelet count < 100 × 10^9^/L [62].

### Management of Direct Oral Anticoagulants During Invasive Procedures

For the prophylaxis of cardioembolism in atrial fibrillation, it is recommended to interrupt DOAC in the hours or days before an invasive procedure and then restart them after a variable amount of time, depending on intervention-related and intrinsic bleeding risk and renal function [16]. This guidance is based on the predictable pharmacokinetics of DOAC, as well as on data showing that bleeding is more likely if heparin is temporary substituted for DOAC or VKA (so called heparin bridging) [63]. It is yet to be proved that this strategy is also valid for DOAC therapy of CAT, even though it is reasonable that this is indeed the case.

## Guideline Recommendations for Treatment and Prevention of Cancer-Associated Venous Thromboembolism

The key indications for treatment and prevention of CAT published by major scientific societies since 2019 are summarized in Table 2 [64.••••, 65.••••, 66.••••, 67.••••, 68.••••].

**Table 2:** Key recommendations of the latest guidelines for treatment and prevention of cancer-associated venous thromboembolism

Recommendations for treatment of CAT	
European Society of Cardiology (ESC) 2022 (64)	Edoxaban, apixaban, or rivaroxaban are recommended for treatment of symptomatic or incidental VTE in patients without contraindications (class I, level A)
International Initiative on Thrombosis and Cancer (ITAC) 2022 (65)	Edoxaban, apixaban, or rivaroxaban are recommended for the treatment of VTE in patients with creatinine clearance ≥ 30 mL/min and in the absence of high risk of gastrointestinal or genitourinary bleeding, strong drug–drug interactions, or gastrointestinal absorption impairment (grade 1A) Treatment of established VTE should last ≥ 6 months (grade 1A); thereafter, termination or continuation of anticoagulation should be based on individual evaluation of the benefit-risk ratio, tolerability, drug availability, patient preference, and cancer activity (guidance in the absence of data)
American Society of Hematology (ASH) 2021 (66)	For short-term treatment of VTE (3–6 months), edoxaban, apixaban, or rivaroxaban are suggested over LMWH (conditional recommendation, low certainty in the evidence of effects + + / + + + +) and VKA (conditional recommendation, very low certainty in the evidence of effects + / + + + +) For long-term anticoagulation (6 months), DOAC or LMWH are suggested (conditional recommendation, very low certainty in the evidence of effects + / + + + +)
National Comprehensive Cancer Network (NCCN) 2020 (67)	DOAC are recommended for treatment of VTE (grade 1)
American Society of Clinical Oncology (ASCO) 2019 (68)	Edoxaban and rivaroxaban are treatment options for VTE (evidence quality: high; strength of recommendation: strong) Anticoagulation beyond the initial 6 months should be offered to selected patients, such as those with metastatic disease or those receiving chemotherapy (evidence quality: low; strength of recommendation: weak to moderate) For long-term anticoagulation, edoxaban, rivaroxaban, or LMWH are preferred over VKA (evidence quality: high; strength of recommendation: strong)
Recommendations for primary prophylaxis	
ESC 2022 (64)	Prophylaxis with apixaban, rivaroxaban, or LMWH may be considered for ambulatory patients at high risk of thrombosis receiving systemic therapy, if there are no significant contraindications (class IIb, level B)
ITAC 2022 (65)	Prophylaxis with apixaban or rivaroxaban is indicated in ambulatory patients with locally advanced or metastatic pancreatic cancer treated with systemic anticancer therapy, who have a low risk of bleeding (grade 1 B) Prophylaxis with apixaban or rivaroxaban is recommended in ambulatory patients who are receiving systemic anticancer therapy and are at intermediate to-high-risk of VTE, identified by a validated risk assessment model (i.e., a Khorana score ≥ 2), and not actively bleeding or not at a high risk for bleeding (grade 1B)
ASH 2021 (66)	Prophylaxis with apixaban or rivaroxaban is suggested for ambulatory patients at high risk for thrombosis receiving systemic therapy (conditional recommendation, moderate certainty in the evidence of effects + + + / + + + +)
NCCN 2020 (67)	Consider apixaban or rivaroxaban for up to 6 months in high-risk patients (Khorana score ≥ 2) starting a new chemotherapy regimen (grade 2A)

*DOAC* direct oral anticoagulants; *LMWH* low-molecular-weight heparin; *VKA* vitamin K antagonists; *VTE* venous thromboembolism

Rivaroxaban, edoxaban, and apixaban have been added as therapeutic options for VTE in cancer patients after the presentation of the results of the landmark RCTs, and currently they are the first choice. With variable emphasis, guidelines advocate caution in using DOAC in subjects with gastrointestinal malignancies, especially of the upper gastrointestinal tract, or when significant interactions are expected with other drugs. DOAC or LMWH are suggested for prolonged anticoagulation beyond 6 months, after careful assessment of potential benefits and risks.

All guidelines propose primary prophylaxis with apixaban or rivaroxaban in patients at high risk of thrombosis, but not of bleeding, receiving systemic anticancer therapy. The susceptibility to CAT is mostly defined by a Khorana score ≥ 2, but also by specific cancer types, such as advanced pancreatic cancer.

## Conclusions

Treatment and to a lesser extent prevention of CAT now rely on DOAC, because of the ease of use and the efficacy-safety profile of these drugs. It has also pointed out that DOAC are cost-effective and cost-saving as compared to LMWH in treating VTE [69].

However, there may be subjects for whom anticoagulation with LMWH is better than with DOAC, such as those with upper gastrointestinal cancer or taking anti-neoplastic agents strongly interfering with DOAC metabolism. LMWH, rather than DOAC, is also desirable for CAT associated with thrombocytopenia < 50 × 10^9^/L.

Furthermore, additional studies are needed to inform the optimal use of DOAC in special situations or patient populations, such as in the proximity of invasive procedures or in individuals with intracranial tumors.
